# The role of oxidative stress in ovarian aging: a review

**DOI:** 10.1186/s13048-022-01032-x

**Published:** 2022-09-01

**Authors:** Fei Yan, Qi Zhao, Ying Li, Zhibo Zheng, Xinliang Kong, Chang Shu, Yanfeng Liu, Yun Shi

**Affiliations:** Dongzhimen Hospital, Beijing University of Chinese Medicine, Beijing, People’s Republic of China

**Keywords:** Ovarian aging, Oxidative stress, Antioxidant, Apoptosis, Inflammation, Cigarette smoking, Melatonin, Vitamin C, Nuclear factor E2-related factor 2

## Abstract

Ovarian aging refers to the process by which ovarian function declines until eventual failure. The pathogenesis of ovarian aging is complex and diverse; oxidative stress (OS) is considered to be a key factor. This review focuses on the fact that OS status accelerates the ovarian aging process by promoting apoptosis, inflammation, mitochondrial damage, telomere shortening and biomacromolecular damage. Current evidence suggests that aging, smoking, high-sugar diets, pressure, superovulation, chemotherapeutic agents and industrial pollutants can be factors that accelerate ovarian aging by exacerbating OS status. In addition, we review the role of nuclear factor E2-related factor 2 (Nrf2), Sirtuin (Sirt), mitogen-activated protein kinase (MAPK), protein kinase B (AKT), Forkhead box O (FoxO) and Klotho signaling pathways during the process of ovarian aging. We also explore the role of antioxidant therapies such as melatonin, vitamins, stem cell therapies, antioxidant monomers and Traditional Chinese Medicine (TCM), and investigate the roles of these supplements with respect to the reduction of OS and the improvement of ovarian function. This review provides a rationale for antioxidant therapy to improve ovarian aging.

## Introduction

Aging is an irreversible physiological and pathological phenomenon in normal metabolism [1]. Aging is complex and multiple factors are known to contribute towards the overall aging process and phenotype [2]. In the female body, the ovary acts as a natural biological clock, thus controlling the process of aging [3]. Ovarian aging refers to the process of gradual decline and eventual exhaustion of ovarian function.

Two major categories of ovarian aging have been identified: physiological and pathological. Physiological ovarian aging refers to the process by which ovarian function deteriorates naturally with age until menopause. Pathological ovarian aging refers to the premature decline of ovarian function, including diminished ovarian reserve (DOR), premature ovarian insufficiency (POI), and poor ovarian response (POR) in the field of in vitro fertilization-embryo transfer (IVF-ET). Pathological aging can be caused by a variety of pathogenic factors [4]. Ovarian aging is characterized by menstrual disorders (amenorrhea or oligomenorrhea) and reduced reproductive function, accompanied by elevated levels of gonadotropins and decreased levels of estrogen. In terms of pathology, ovarian aging predominantly manifests as a reduction in the number of follicles and a decline in the quality of oocytes [5]. The terminal stage of ovarian aging is menopause. Following menopause, dysfunction of the endocrine, cardiovascular and nervous systems, among others, becomes apparent or aggravated with hormonal changes in female body. Therefore, ovarian aging is considered an important node in the process of female aging.

As a classic theory of aging, free radical theory was first proposed in the 1950s. The important role of free radical has been extensively studied across multiple diseases and is considered a key mechanism of ovarian aging. Free radicals are a high-activity pro-oxidation group of molecules produced during aerobic metabolism, including reactive oxygen species (ROS) and reactive nitrogen species (RNS) [6]. ROS is primarily a byproduct of mitochondrial oxidative phosphorylation. A temperate amount of ROS is essential for the normal physiological functions of the body. ROS are not only involved in the synthesis of active substances, cellular detoxification and immune function [7], they also act as an important second messenger that participates in the intercellular signal transduction and regulation of gene expression, thereby maintaining cellular homeostasis [8].

Under physiological conditions, the oxidative and antioxidant systems are in dynamic equilibrium. The antioxidant system is composed of both enzymes and non-enzymes. Of these, the key enzymes include superoxide dismutase (SOD), catalase (CAT), glutathione (GSH) and glutathione peroxidase (GSH-Px). Non-enzymes mainly include melatonin, vitamins (C, E) and trace elements (copper, zinc and selenium). However, when ROS are overproduced or antioxidant utilization increases, this leads to an imbalance of redox reactions and induces an oxidative stress (OS) state in the body [9]. Extensive studies have shown that the OS state of the ovarian microenvironment can result in pathological damage, including meiotic arrest in oocytes, granulosa cell apoptosis, and corpus luteum dysfunction, thus accelerating the process of ovarian aging. Supplementation with antioxidants can improve the ovarian OS state and enhance ovarian function. This article reviews research progress in the field of OS in relation to ovarian aging.

## The physiological role of ROS in the ovary

In the ovary, ROS are involved in the regulation of oocyte growth, meiosis, ovulation and other physiological processes [10]. During follicular growth, increased steroid production leads to the expression of cytochrome P450, thus resulting in ROS formation. Meanwhile, the increased secretion of estradiol (E_2_) in growing follicles triggers the expression of peroxidase CAT, resulting in a dynamic balance between ROS and antioxidants [11]. The precise regulation of meiotic arrest and recovery of oocytes is essential for female reproductive development. ROS stimulation regulates the progress of meiosis I; in contrast, the progression of meiosis II is mainly controlled by antioxidants in the ovary. This demonstrates the complex relationship between free radicals and antioxidants within the meiotic maturation process of oocytes [12]. The surge of luteinizing hormone (LH) prior to ovulation increases the levels of inflammatory precursors in the ovary, thus leading to the excessive production of ROS. Increased ROS induces apoptosis in the granulosa cells, which further leads to follicular wall rupture and ovulation; therefore, this is regarded as an important ovulation signal [13]. Similarly, the regression of the corpus luteum is also mediated by OS-induced apoptosis of luteinized granulosa cells [14]. The balance of ROS is also critical within the in vitro setting, and can exert influence on oocyte maturation, fertilization, and subsequent embryo implantation and development [15]. The appropriate concentration of ROS in follicular fluid is not only an indicator of good follicular metabolic activity but can also be used as a potential marker for predicting the outcome of IVF-ET [16].

## Pathological mechanisms related with OS in ovarian aging

### Apoptosis

Apoptosis has been studied extensively in ovarian aging [17, 18]. Apoptosis in ovarian cells can cause extensive follicular atresia or regression and is considered to be one of the most important mechanisms underlying ovarian aging [19]. OS has been shown to induce apoptosis in ovarian cells by various processes, including exogenous pathways, endogenous pathways and by endoplasmic reticulum stress (ERS) [20–22]. In the exogenous pathway, excess ROS in ovarian tissue can activate the Fas/FasL pathway and recruit caspase-8 (CASP8) to form a death-inducing signaling complex (DISC) with Fas and FasL. Activated CASP8 then activates CASP3, CASP6, CASP7 and cleaves various downstream intracellular substrates that are necessary for cell survival, thereby inducing apoptosis [23]. In the endogenous pathway, ROS can disrupt mitochondrial homeostasis in ovarian cells, thus resulting in the release of Cytochrome C from the mitochondria. Cytochrome C and apoptotic protease-activating factor (Apaf-1) form a multimeric complex, continue to activate CASP3 and CASP9, and upregulate the Bax/Bcl-2 ratio, thus inducing apoptosis [24]. In addition, OS can activate inositol-requiring enzyme 1 (IRE1) and protein kinase RNA-like ER kinase (PERK), induce unfolded protein response (UPR) factors and ultimately promote apoptosis in granulosa via the ERS pathway [25, 26].

Oocyte apoptosis leads to the loss of germ cells directly. Granulosa cell apoptosis leads to nutrient deprivation in ovarian cells and induces metabolic disorders in the ovarian microenvironment, thus aggravating the decline in ovarian function [27]. Furthermore, OS can lead to apoptosis in female germline stem cells (FGSCs). Phenotypic changes in FGSCs reduce their proliferative capacity and stemness. And the ovary also loses the potential to replenish the primordial follicle pool and produce oocytes, ultimately reducing ovarian reserve [28]. The cell debris generated by apoptosis can continue to affect the ovarian microenvironment. Apoptosis elevated the levels of cell-free DNA in follicular fluid which stimulates the massive production of intracellular ROS and ultimately exacerbates apoptosis [29].

### Inflammation

Long-term chronic inflammation accelerates aging in the body [30]. An abundance of evidence has shown that chronic inflammation is closely related with ovarian aging [31]. Clinical studies have found that the serum levels of IL-6 and IL-21 were significantly higher in POI patients than in healthy women of the same age [32]. NLRP3, as an inflammasome in the NLR family, plays an important role in inflammation [33]. NLRP3 was found to be highly expressed in the granulosa cells of DOR patients [34]. After NLRP3 reduction, the levels of pro-inflammatory cytokines were down-regulated while the levels of AMH and the number of primordial follicles increased [34].

OS is closely linked with inflammation. Excessive levels of ROS in the body can trigger the assembly and activation of the NLRP3 inflammasome [35], which subsequently promotes the infiltration of inflammatory cells and the secretion of the pro-inflammatory cytokines IL-1β and IL-18 in tissues [36]. ROS can also directly activate nuclear factor-k-gene binding (NF-κB) to promote inflammation while activation of NF-κB further upregulates NLRP3 expression [37]. Moreover, after immune cells are activated, intracellular ROS are produced in large quantities to participate in the activation of the immune response, further leading to increased OS damage [38].

OS and inflammation produce synergistic disruptive effects on ovarian tissue. A previous study showed that the levels of the products of OS damage (carbonylated and nitrated proteins) and inflammatory markers (TNF-α, IL-1β) were significantly higher in the ovaries of aged mice than in younger mice [39]. Ovarian RNA transcriptome analysis further revealed that OS-related proteins (Nrf2, SOD2, CAT, GSH-px1) and genes encoding NLRP3 inflammatory vesicles could be used as key biomarkers to differentiate between young and aging mice [40]. A variety of models of functional ovarian damage were shown to exhibit high levels of inflammatory factors and OS markers [41, 42]. Furthermore, the use of antioxidants or dietary supplements (quercetin, ginsenoside Rg1, Vitamin B12) has been shown to significantly improve both inflammation and OS, thus enhancing ovarian function [43–45]. Therefore, inflammation plays an important role in ovarian aging.

### Mitochondria

Mitochondria are important organelles in cells. They can generate ATP through the process of oxidative phosphorylation and are known as the energy production machinery for cells [46]. Mitochondria also have their own genomes that encode polypeptides involved in energy production [47]. Mitochondrial DNA (mtDNA) is vulnerable to ROS attack due to the lack of histone protection and an overlap with the ROS generation site in the mitochondrial inner membrane [48]. Excessive levels of ROS not only induce mtDNA mutations to result in inefficient electron transport chain (ETC) expression, but also mediate abnormal mtDNA-protein cross-linking, thus leading to mitochondrial dysfunction in several ways [49]. Mitochondrial dysfunction further exacerbates the leakage of ROS from the ETC, thereby exacerbating intracellular OS damage [50]. Ultimately, this cascade of amplified injury can have serious adverse effects on ovarian function.

Mitochondria are the most numerous organelles in the oocyte and provide sufficient energy to allow fertilization and maintain embryogenesis [51]. Mitochondria are involved in important processes during oocyte meiosis, including spindle assembly, chromosome segregation and cell maturation [52]. Therefore, the number and distribution of mitochondria, along with alterations of the mtDNA sequence, are closely related to the quality of oocytes and have important impacts on embryonic development [53]. Zhang et al. constructed a secondary oocyte OS injury model and found that the ATP level and mitochondrial membrane potential were both decreased; this was accompanied by spindle damage [54]. Recombinant peroxiredoxin 3 (Prdx3) is localized in the mitochondria and acts as a key regulator of mitochondrial ROS [55]. Global gene expression analysis of aged mouse oocytes revealed reduced Prdx3 mRNA expression and increased sensitivity of oocytes to OS [56].

Granulosa cells represent the largest cell population in the ovary. Their growth, proliferation and division require abundant and stable mitochondria to supply appropriate amounts of energy [57]. During proliferation, granulosa cells experience a significant increase in ROS levels and mtDNA damage [58]. At the same time, granulosa cells exhibit reduced mitochondrial membrane potential and reduced expression levels of mitochondrial-related genes (Nd1, Cytb, Cox1 and ATPase6), ultimately resulting in a poor state of decreased viability and cell cycle arrest [59]. Tanabe et al. reported that the OS damage products 8-OHdG, yH2AX and HEL were significantly elevated and that the proportion of active mitochondria was significantly reduced in an OS injury model of granulosa cells [60]. Mitochondrial dysfunction disrupted the bidirectional interaction between oocytes and granulosa cells, blocking the exchange of important substances such as pyruvate, amino acids and nucleotides, which in turn lead to stagnant cell growth and development [61].

### Telomeres

Telomeres are short repetitive sequences that are located at the ends of eukaryotic chromosomes and mainly composed of non-coding DNA and telomere-binding proteins. The telomeres are responsible for maintaining genome integrity and chromosomal stability [62]. Telomere length gradually shortens with an increasing number of cell divisions and are therefore considered to be closely associated with the degree of aging in the human body [63]. The correlation between telomere status and ovarian function in women has also received increasing levels of attention. Clinical studies have also found that the length of telomeres in the granulosa cells of POI patients is significantly shorter, and that telomerase activity is significantly reduced [64]. Telomere damage was also observed in naturally aged ovarian cells, along with reduced expression levels of telomerase (TERC), telomeric reverse transcriptase (TERT) and telomere-related proteins (TRF1, TRF2, POT1) [65, 66]. The relative length of telomeres in cumulus cells is closely related to oocyte and embryo quality and can be used as a potential marker for screening high-quality oocytes in the field of assisted reproductive technology (ART) [67].

Telomeres lack protective proteins and are therefore highly susceptible to ROS attack and shortening [68]. Sirtuin 6 (Sirt6) is thought to play an important role in the stabilization of telomeres in oocytes [69]. As a trans-activator, Sirt6 participates in the regulation of Nrf2, thus maintaining cellular redox homeostasis [70]. Liao et al. found that both the mRNA and protein levels of Sirt6 were significantly lower in aged oocytes and that this was accompanied by a reduction in telomere length [71]. Ge et al. found that Sirt6-specific depletion in oocytes could exacerbate mitochondrial dysfunction and apoptosis in early embryos [72].

Antioxidant supplementation was previously shown to improve OS and telomere status, thus alleviating ovarian aging. A study by Akino et al. showed that the administration of dimethyl fumarate (DMF) led to the activation of the Keap1/Nrf2 signaling pathway in the ovaries of aged mice [73]. DMF supplementation increased mRNA and protein expression levels of telomerase and increased the number of primordial follicles [73]. In another study, Liu et al. found that long-term supplementation with low doses of NAC increased telomere length, elevated telomerase activity, improved oocyte quality and the number of fertilized oocytes, ultimately increasing litter size [74].

### Biomacromolecules

Excess ROS can result in oxidative damage to intracellular biomacromolecules such as proteins and lipids [75]. Approximately 70% of oxidized molecules in cells are proteins, indicating that proteins are the main targets of ROS attack [76]. OS can lead to multiple modifications of proteins, including tyrosine nitration, sulfonation, thiol oxidation and 4-hydroxynonenal (4HNE) protein adducts [77]. Impaired protein function further leads to abnormal activation or inactivation of the signaling pathways required for normal ovarian physiology [77]. A number of studies have shown that multiple protein OS damage products can be observed in ovarian cells and follicular fluid, including advanced oxidation protein products (AOPPs), carbonylated and nitrated proteins, 4-HNE [78–81]. Follicular membranes contain large amounts of unsaturated fatty acids which are susceptible to ROS attack, thus leading to lipid peroxidation (LPO). LPO leads to the formation of acrolein and malondialdehyde (MDA) which can lead to a further increase in ROS production [82]. Oocyte meiosis and granulosa cell proliferation require an adequate supply of protein, carbohydrate and lipid; however, OS can directly damage macromolecular structures and therefore lead to ovarian damage [83].

## Factors related to OS that lead to ovarian aging

### Aging

Age is the most important intrinsic factor affecting ovarian function and fertility [84]. With increasing age, the number of primordial follicles decreases exponentially, while the frequency of aneuploidy in oocytes increases, eventually leading to a significant reduction in pregnancy and live birth rates [85]. Clinical studies have shown that a woman’s fertility declines linearly by approximately 10% per year after the age of 35, with only 1% fertility by the age of 43 [86].

Aging leads to increased ROS production and reduced activity of antioxidant systems, which together lead to increased intracellular OS damage. Accumulation of DNA damage is one of the key factors in damage of oocyte quality with age [87]. DNA damage accumulation increases chromosomal fragmentation and affects meiosis, spindle assembly and mitochondrial distribution in the oocyte, ultimately affecting embryo development [88]. Prolonged quiescence during meiosis I renders oocytes unusually sensitive to the accumulation of DNA damage [89].

DNA damage occurs frequently in aged oocytes, such as DNA double-strand breaks (DSBs), however, DNA damage response (DDR) repair is inefficient [90]. Studies have confirmed that the expression levels of oocyte DNA repair genes, such as BRCA1, MRE11, and H2AX, decrease significantly with age [91, 92]. BRCA1 recruits various DNA repair proteins to promote the homologous recombination (HR)-based pathway for DNA double-strand break repair (DSBR), and therefore plays an important role in DNA repair in aging ovaries [93, 94]. In addition, BRCA1 is required to prevent abnormal chromosome segregation [95]. Genome-wide association study (GWAS) analysis confirmed that genes associated with DDR, particularly BRCA1, are key determinants of the natural menopausal age in women [96].

Mitochondrial dysfunction due to aging has also been found to be a fundamental factor in the decline of oocyte quality. Mitochondrial dysfunction increases ROS leakage and mtDNA mutations and reduces ATP synthesis, thus affecting meiosis and decreasing oocyte quality [56]. In addition, biomacromolecular damage also accumulates in ovarian cells with age; this represents another cause of ovarian hypofunction [97].

### Cigarette smoking

The extensive harm of cigarette smoking to the human body is well established. Smoking has been demonstrated to damage ovarian function and closely related with infertility, pregnancy complications and fetal abortion [98]. Tobacco smoke contains stable pro-oxidants that can directly increase ROS in the body [99]. Tobacco also contains harmful chemicals such as nicotine and tar that can deplete protective antioxidants, ultimately leading to an OS state [100].

Researchers collected follicular fluid from female patients undergoing ART. High levels of tobacco metabolites were found in the follicular fluid of women who smoked, along with increased levels of lipid peroxidation damage and decreased levels of antioxidants [101, 102]. A meta-analysis of 12 studies reported that time to conception, the incidence of infertility, and the number of IVF treatment cycles, were significantly higher in smokers than in non-smokers; this was associated with ovarian OS damage caused by tobacco exposure [98].

The extent of ovarian OS damage is thought to fluctuate with the amount and duration of smoke exposure [103]. Any level of smoke exposure cannot be considered safe, either passively or only at low levels [104]. This raises a warning for women of childbearing age who have a smoking habit or are often passively exposed to cigarette smoke. Moreover, the harm caused by cigarette smoking can also impair ovarian function in female offspring of smokers. Studies investigating the ovaries of female offspring (F1 generation) of smoking mothers showed significantly increased OS levels in oocytes, further leading to apoptosis and the abnormal proliferation of granulosa cells [105]. This effect continued into the F2 generation with impact on ovarian function not declining until the F3 generation [106].

### High-sugar diet

High-sugar diets are generally considered to be an unhealthy lifestyle [107]. Abundant clinical studies have confirmed that a high-sugar diet can lead to an OS state in the body, which accelerates the development of multisystem pathologies, such as diabetes, neurodegenerative and cardiovascular diseases [108–110]. A high-sugar diet, whether long-term, short-term or fluctuating, can affect the body’s redox status and thus have a negative impact on ovarian function [111].

Excess carbohydrates in the body combine with proteins to form advanced glycation end products (AGEs) [112]. AGEs bind to specific cell surface receptors (RAGE) and promote ROS production via NF-κB and NADPH oxidase [113]. RAGE proteins are abundantly distributed on the membranes of oocytes, stromal cells and granulosa cells. Therefore, prolonged exposure to high concentrations of AGEs can lead to a gradual accumulation of OS damage in the ovary [114].

In addition, ROS are involved in a key step in the modified advanced glycation in AGE production. Thus, the accumulation of AGEs and the increase of ROS form a positive feedback loop that together increase OS [115]. Both AGEs and ROS can interfere with insulin signaling pathways and affect the normal function of ectopic glucose transporters. And this leads to a reduction in the uptake of glucose by ovarian cells, resulting in poor follicle development and an acceleration of ovarian aging [116]. OS damage induced by AGEs may also induce inflammation and hypoxia, damaging the blood vessels of the ovaries and further accelerating ovarian aging [116]. In addition, AGEs can directly stimulate the production of extracellular matrix (ECM) and the abnormal cross-linking of collagen and elastin in the ovary, thus affecting the proliferation and division of granulosa cells and ultimately disrupting ovarian function [117].

### Pressure

Females are frequently exposed to multiple forms of pressure, including career, family and childbirth [118]. Both repeated acute stress and long-term persistent psychological stress have been shown to be independent risk factors for pregnancy rates, live birth rates, preterm delivery and low birth weight [119].

Pressure can impair ovarian function in many ways. On the one hand, pressure can induce the release of the stress hormone cortisol, which acts on the hypothalamic–pituitary–adrenal (HPA) and hypothalamic-pituitary-ovarian (HPO) axes to elicit direct negative effects on the ovary [120]. At the same time, the body’s adaptation to pressure leads to a dramatic rise in intracellular OS and an endogenous burst of internal calcium stores [121]. These trigger multiple regulatory mechanisms in ovarian cells, such as autophagy, apoptosis and paraptosis, further leading to cell cycle arrest and a decline in ovarian function [122, 123]. On the other hand, cortisol also reduces the secretion of antioxidants such as estradiol-17β, thus leading to decreased OS defense in ovarian cells [124]. Zhao et al. investigated the effect of chronic unpredictable mild stress (CUMS) on ovarian function in rats. The results showed that the CUMS group had increased ovarian ROS levels, mitochondrial dysfunction and cell apoptosis, along with decreased AMH and E_2_ levels. [125, 126]. In addition, pressure has been found to decrease levels of nerve growth factor (NGF) and brain-derived neurotrophic factor (BDNF) [127, 128]; thus leading to over-activation of the sympathetic nerves in the ovary, thereby impairing ovarian function [129].

### Superovulation

Controlled ovarian hyperstimulation (COH) is widely used in IVF-ET to obtain a sufficient number of oocytes for downstream processing [130]. However, successful ovulation is accompanied by a rapid increase in OS levels and inflammatory response [131]; thus, whether COH can lead to ovarian OS damage has received widespread attention.

Clinical studies have identified significantly lower levels of antioxidants (including alpha-tocopherol, TAA and paraoxonase) in the follicular fluid of women receiving COH intervention when compared to women receiving natural cycle (NC) intervention [132]. Women who receive COH also exhibited an abnormal inflammatory response [133, 134]. OS damage to the ovaries was also significantly correlated with COH cycles. Higher markers of OS damage and lower antioxidant enzymes in the ovaries could be observed in cycles 3–5 when compared to cycles 1–2. This was accompanied by lower quality embryo rates, implantation rates and clinical pregnancy rates [135].

Similarly, related experimental studies have confirmed the oxidative damage to the ovaries by superovulation. Nie et al. performed continuous superovulation intervention in mice and observed a significant increase in OS damage products and oocyte apoptosis in the super-promoting group that was associated with activation of the Sirt1/FoxO1 signaling pathway [136]. Repeated ovulation promotion increased the number of abnormal mitochondria in mouse oocytes, reduced the volume of the primordial follicular pool, decreased serum AMH levels and significantly inhibited embryo development [137]. This process also significantly increases the risk of long-term complications such as osteoporosis and cardiovascular disease [138]. In contrast, oral contraceptives have been shown to alleviate age-related ovarian aging and fertility decline by suppressing ovulation [139].

### Chemotherapy

The application of chemotherapy agents has increased the chances of long-term survival for cancer patients. However, the damage that these agents cause to ovarian function has received increasing levels of attention [140]. Chemotherapy not only induces atresia of growing follicles leading to temporary amenorrhea [141], but also accelerates overactivation of the primordial follicular leading to a reduced ovarian reserve [142]. Multiple clinical trials have identified varying degrees of impaired ovarian function in women who have received chemotherapy [143–145].

OS damage has been identified as an important mechanism by which chemotherapy side effects occur [146]. Cisplatin is commonly used to treat solid tumors. Researchers gave rats an intraperitoneal injection of cisplatin and found that the expression levels of 8-OHdG and MDA increased significantly, while the levels of antioxidant enzymes SOD and GSH-Px decreased significantly in the ovaries [147]. At the same time, the ovarian cortex was significantly damaged or was even indistinguishable from the medulla [146]. Paclitaxel is often used in combination with carboplatin as a chemotherapy regimen for non-small cell lung cancer and ovarian cancer. Qin et al. found that paclitaxel intervention in mice resulted in a significant increase in the lipid peroxidation product 4-HNE and apoptosis in the ovary; this was accompanied by a reduced number of follicles, along with morphological abnormalities of follicles at all stages [140]. Methotrexate (MTX) is commonly used to treat tumors and autoimmune diseases. MTX has been shown to deplete NADPH and antioxidant enzymes and to induce mitochondrial dysfunction, DNA breaks and disruption of the spindle assembly, thereby affecting oocyte maturation [148]. MTX could also cause severe follicular degeneration and intra-ovarian hemorrhage and edema, ultimately destroying ovarian function [149].

### Industrial pollution

With the rapid development of industrialization, many industrial by-products have been shown to affect ovarian function by increasing ROS levels. Bisphenol A (BPA) is an important material used in the synthesis of polycarbonate and epoxy resins. BPA was found to increase ROS levels in ovarian cells in a dose- and time-dependent manner, reduce mitochondrial membrane potential, and activate the JNK signaling pathway to accelerate granulosa cell apoptosis [150]. Fluorene 9-bisabolol (BHPF), as a substitute for BPA, also poses a risk to ovarian function. Following BHPF intervention, oocytes were found to exhibit increased levels of ROS and DNA damage, accompanied by mitochondrial dysfunction, polar body extrusion and disruption of the spindle assembly [151]. Nonylphenol (NP) is an important raw material for the fine chemical industry. Related studies have shown that NP can increase ROS levels in rat ovarian granulosa cells, further activating the AKT/AMPK/mTOR signaling pathway, triggering excessive cellular autophagy and ultimately reducing ovarian function [152]. Di-phthalate (DEHP) is a type of common plasticizer and has been found to increase intracellular β-galactosidase (β-gal) activity, activate the Bax/Bcl-2 signaling pathway and CASP3 expression, and inhibit steroid synthesis, ultimately leading to premature ovarian aging [153].

**Fig. 1:**
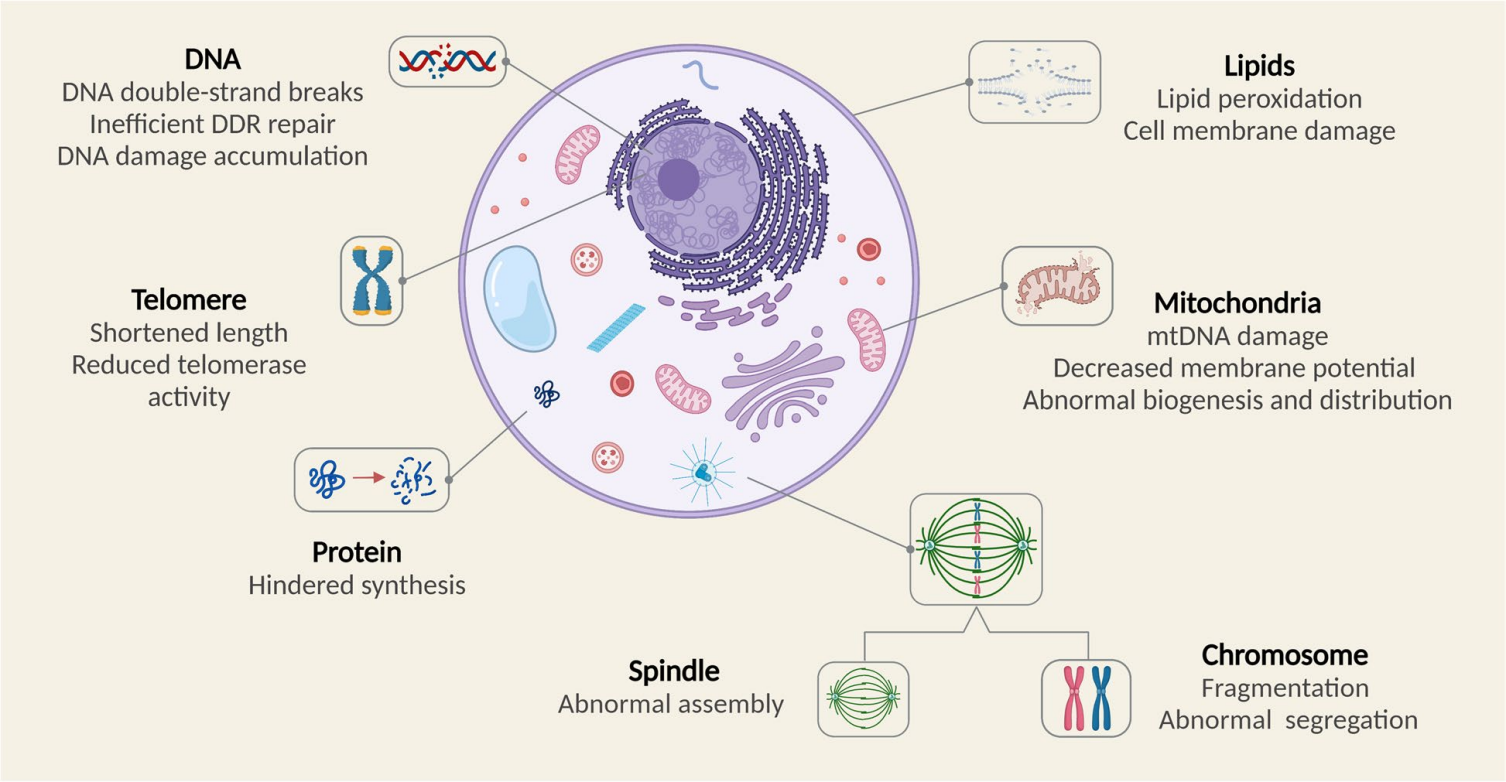
Intracellular OS damage

**Fig. 2:**
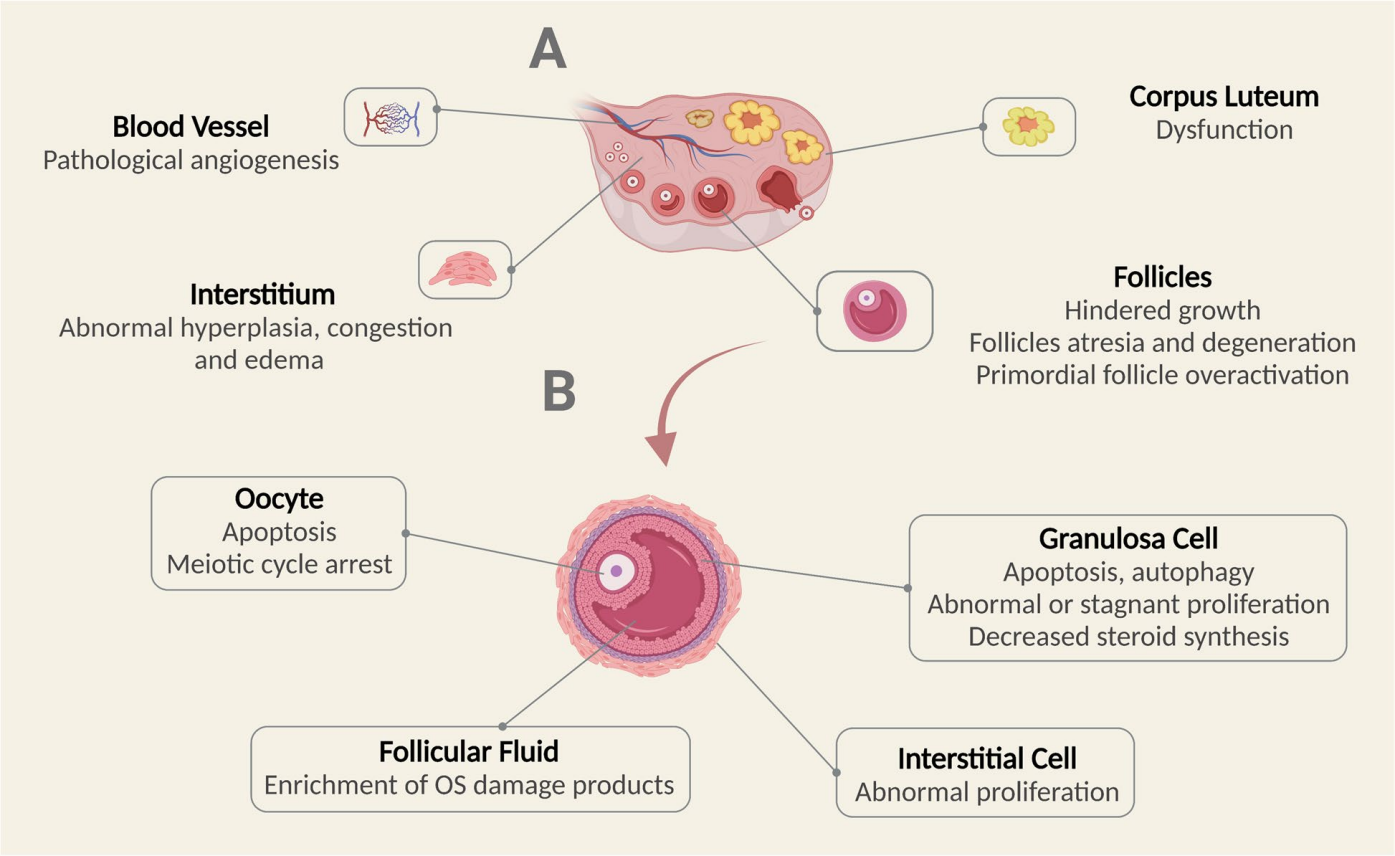
OS injury in the ovary. **A** OS damage of blood vessels, interstitium, follicles and corpus luteum in the ovary. **B** OS damage of oocytes, granulosa cell, ovarian interstitial cell and follicular fluid in ovarian follicles

In conclusion, a variety of external factors can accelerate ovarian aging by increasing the OS state in the body. External factors include lifestyle choices such as smoking, high-sugar diets, and excessive psychological pressure; superovulation during ART, chemotherapy drugs and industrial environmental pollutants are also important factors, as summarized in Table 1. An increased OS state can cause cellular damage, including DNA damage, mitochondrial dysfunction and biomacromolecular damage. OS damage in cells can lead to the meiotic arrest of oocytes, the inhibition of granulosa cell proliferation, and the abnormal proliferation of interstitial cells, all of which can accelerate the ovarian aging process, as demonstrated in Figs. 1 and 2.

**Table 1:** Related OS factors leading to ovarian aging

Influence Factors	Injury Effects Summary
Aging	• Decreased pregnancy and live birth rates • Increased ROS production, decreased antioxidant system defense • Frequent DSBs, inefficient DDR repair, accumulation of DNA damage • Mitochondrial dysfunction • Accumulation of biomacromolecules damage
Cigarette smoking	• Extension of conception time, reduction of fertility incidence, extension of IVF-ET treatment cycles • Increased ROS formation, depletion of protective antioxidants • Impairment of ovarian function in female offspring
High-sugar diet	• Increasing levels of ROS through AGEs production • Dysregulation of insulin signaling pathway • Hyperplasia of extracellular matrix • Impairment of blood vessel • Induction of inflammation and hypoxia
Pressure	• Reduced pregnancy rate • Direct negative effects on HPO and HPA axes • Decrease of antioxidant expression • Accelerated cellular autophagy, apoptosis and paraptosis • Disorder of endocrine hormone
Superovulation	• Increased ROS levels and inflammatory response • Reduced primordial follicles • Increased risk of osteoporosis and cardiovascular disease in the long term
Chemotherapy	• Depletion of antioxidant enzymes • Mitochondrial dysfunction, lipid peroxidation • Exacerbated cell apoptosis • Atresia of growing follicles, overactivation of primordial follicular
Industrial Pollution	• Reduction of mitochondrial membrane potential • Exacerbated DNA damage • Spindle assembly destruction • Increased cell apoptosis and autophagy • Inhibition of steroid synthesis

*ROS* Reactive oxygen species, *DSBs* DNA double-strand breaks, *DDR* DNA damage response, *IVF-ET *In vitro fertilization-embryo transfer, *AREs* Antioxidant response elements, *HPO* Hypothalamic-pituitary-ovarian, *HPA* hypothalamus–pituitary–adrenal

## OS-related signaling pathways in ovarian aging

**Fig. 3:**
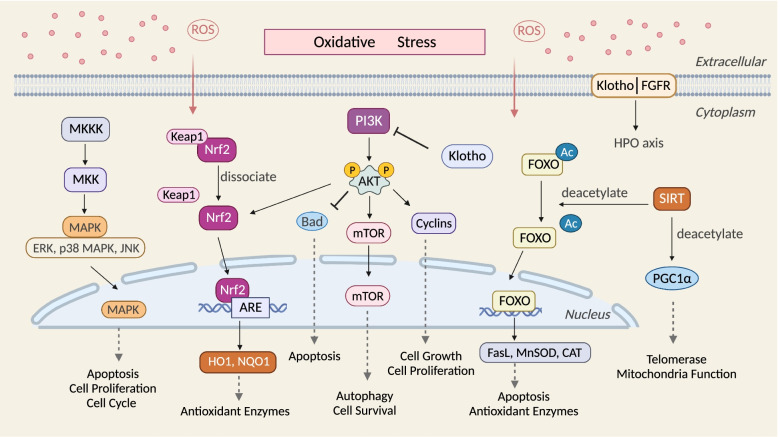
OS-related signaling pathways in ovarian aging. Excess levels of ROS promote the dissociation of the Keap1-Nrf2 complex and Nrf2 translocation into the nucleus to bind to AREs, thus promoting the expression of antioxidant enzymes. Sirt can deacetylate key proteins involved in the cellular stress response such as FoxO, and regulate both telomerase activity and mitochondrial function through PGC1α. The MAPK cascade signaling pathway is activated by ROS to deliver extracellular signals to the nucleus, promote apoptosis, inhibit proliferation and induce cell cycle arrest. AKT plays an important role in the regulation of cellular redox homeostasis, and phosphorylated AKT can regulate a variety of downstream proteins (Bad, mTOR, Cyclins and Nrf2) to further regulate cellular apoptosis, autophagy and proliferation. FoxO senses cellular OS status and acts as a transcription factor to regulate cell apoptosis and the expression of antioxidant enzymes. Klotho regulates cellular oxidative homeostasis through the PI3K/AKT pathway, and the HPO axis through the FGF-Klotho endocrine system

Excessive ROS can result in crosstalk between a series of signaling pathways and protein factors within the body. This section focuses on signaling pathways related to OS and ovarian function, including Nrf2, Sirtuins, MAPK, AKT, FoxO family and Klotho signaling pathways, as demonstrated in Fig. 3.

### Nrf2 signaling pathway

Nuclear factor-E2-related factor 2 (Nrf2) is a transcription factor involved in regulating antioxidant responses to protect cellular functions [154]. Under normal conditions, Nrf2 binds to Kelch-like ECH-associated protein 1 (Keap1), leading to the ubiquitination and degradation of Nrf2. This facilitates only a basal level of antioxidant enzyme expression. However, in response to cellular OS, the Nrf2-Keap1 complex dissociates and Nrf2 is translocated to the nucleus, thus leading to the rapid accumulation of nuclear Nrf2. Nuclear Nrf2 binds genomic antioxidant response elements (AREs) to promote the gene expression of a series of target genes including antioxidant enzymes and detoxification factors, such as heme oxygenase-1 (HO-1), NAD(P)H: quinone-oxidoreductase (NQO1), and glutamate-cysteine ligase subunit catalysis (GCLC) [155]. Lead exposure in mice inhibited the Keap1/Nrf2 pathway and exacerbated ovarian OS damage, which inhibited oocyte maturation and fertilization [156]. Compared to wild-type mice, Nrf2^−/−^ mice exhibited premature follicular activation and thus an age-dependent decline in ovarian function [157]. Loss of Nrf2 function also blocked microsomal epoxide hydrolase expression and reduced cellular antioxidant capacity, thereby increasing ovarian sensitivity to environmental pollutants [158, 159].

Related antioxidant treatment may also improve ovarian OS status by modulating the Nrf2 pathway. The oral administration of DMF intervention to mice was shown to upregulate ovarian tissue antioxidant enzyme and telomere protein expression, increase serum AMH level, slow down DNA damage accumulation, and protect the primordial follicular pool through the Nrf2 pathway [73]. In vitro experiments further revealed that the antioxidant epicatechin could protect granulosa cells from OS damage by activating the Nrf2 signaling pathway, upregulating NQO1, HO-1 and SOD expression [160]. Therefore, the Nrf2 signaling pathway is one of the most important defense mechanisms for cells to resist OS damage.

### Sirt signaling pathway

Sirtuins belong to the class III nicotinamide adenine dinucleotide NAD + dependent deacetylase family which included seven subtypes; these proteins are involved in many physiological functions in cells [161]. Of these, Sirt1 is a key regulatory protein of cellular metabolism and OS and has been extensively studied in ovarian function [162].

Sirt1 can deacetylate key proteins involved in the cellular stress response, such as FoxO, causing the upregulation of antioxidant enzymes such as CAT and GSH-Px1 [163]. Previous research showed that consecutive superovulation could exacerbate OS damage in the ovary, increase granulosa cell apoptosis and decrease oocyte quality and primordial follicle number through the Sirt1/FoxO1 signaling pathway [136]. Sirt1 was shown to be involved in the first-line defense against ROS via the FoxO3a-MnSod axis in the germinal vesicle phase (GV) of immature mouse oocytes [164]. Sirt1 deletion in oocytes led to reduced oocyte quality, the inhibition of oocyte division, and increased OS damage during the embryonic period, ultimately causing negative effects on pregnancy outcome [165]. The knockdown of Sirt1 in granulosa cells was found to impair E_2_ synthesis and secretion and significantly reduce the expression of E_2_-related receptors (ESR, FSHR and AMHR2) [166]. In addition, Sirt1 mediates the activation of peroxisome proliferator-activated receptor gamma coactivator l-alpha (PGC1α), further promoting mitochondrial biogenesis and oxidative phosphorylation during primordial follicle activation [167]. Therefore, Sirt1 has an important role in the antioxidant protection of oocytes, granulosa cells and early embryos.

Other subtypes of the Sirtuins family also play a role in the protection of ovarian function. Sirt2 controlled histone H4K16 deacetylation and is therefore a key effector of oocyte meiosis [167]. Sirt3 can regulate the expression of aromatase and 17-hydroxysteroid dehydrogenase, and promote progesterone secretion [168]. Sirt6 has been shown to prevent OS-induced DNA damage and play an important role in stabilizing oocyte telomeres [79].

### MAPK signaling pathway

The mitogen-activated protein kinase (MAPK) pathway is an important transmitter that mediates extracellular signals from the cell membrane surface to the nucleus [169]. The MAPK signaling pathway consists of MAP kinase kinase kinase (MKKK), MAP kinase kinase (MKK) and MAPK. Tertiary kinases are activated sequentially and participate in internal and external reactions to further regulate cell proliferation, differentiation, survival and death [170].

Extracellular regulated protein kinases (ERK), p38 MAPK and c-Jun N-terminal kinase (JNK) are all part of the MAPK family and have been extensively studied in ovarian aging [171]. Sun et al. demonstrated that CUMS could exacerbate ROS levels in mouse ovaries, inhibit granulosa cell proliferation and accelerate cellular senescence via the MAPK pathway [172]. The ERK pathway was shown to be involved in autophagy-related inhibition of cell proliferation and cellular senescence, while the JNK and p38 MAPK pathways have been shown to be associated with cell cycle arrest [172]. Apoptosis signal-regulating kinase 1 (ASK1) is a widely expressed redox-sensitive mitogen/threonine kinase. Upon cellular OS injury, ASK1 was activated and further activated JNK, thereby inducing downstream signaling [173]. Exposure to bisphenol AF can regulate the ROS-ASK1-JNK pathway, reduce mitochondrial membrane potential, and induce apoptosis in ovarian granulosa cells [174].

Antioxidant supplementation can reduce the damaging effects of ROS on the ovaries through the MAPK pathway. Melatonin can inhibit mROS production and increase antioxidant enzyme expression through the ERK pathway to further improve excessive autophagy induced G2/M cell cycle arrest [175]. NAC was shown to be able to attenuate OS damage in ovarian granulosa cells by advanced oxidation protein products (AOPPs) via the JNK/p38 MAPK-p21 pathway, ultimately improving follicular atresia [176].

### AKT signaling pathway

Serine/threonine kinase AKT is the central survival mediator in cell signaling transmission and is activated by a variety of stimuli including ROS, growth factors and cytokines [177]. Phosphorylated AKT can regulate a variety of downstream proteins, such as Bad, mTOR and Cyclins, thus playing an important role in various biological processes such as apoptosis, autophagy and proliferation [178].

Bad is a Bcl-2 homology domain 3-related protein involved in apoptosis. AKT can regulate Bad activity by phosphorylating at Ser-136 [179]. Dephosphorylated Bad was able to form a pro-apoptotic complex with anti-apoptotic factors Bcl-2 or Bcl-xL, which activated pro-apoptotic members such as Bax or Bak, and jointly promoted cell apoptosis [180]. Studies have shown that H_2_O_2_ can inhibit the PI3K/AKT pathway, and induce the elevated expression of Bad, Bax and CASP9, further accelerating granulosa cell apoptosis [181]. Meanwhile, OS can also increase the expression of p53 up-regulated modulator of apoptosis (PUMA) through the PI3K/AKT pathway and induce apoptosis in another way [182]. mTOR is a serine/threonine protein kinase in the PI3K-related kinase (PIKK) family. AKT blocks the negative regulation of small G protein Rheb by TSC1/2 and indirectly activates mTOR complex 1 (mTORC1) which is involved in the critical regulation of cellular autophagy [183]. OS injury initiates autophagy by inhibiting the PI3K/AKT signaling pathway, promoting the dissociation between mTORC1 and ULK1, further phosphorylating autophagy-related proteins such as Atg13 [184]. It was found that H_2_O_2_ intervention in granulosa cells inhibited the PI3K/AKT/mTOR pathway and increased LC3-II/LC3-I and Beclin-1 expression [185]. Activation of AKT regulates the cell cycle by regulating the function of cell cycle proteins (cyclins). Intervention of isorhamnetin reduced cellular OS level, and increased the expression of Cyclin D, Cyclin E and Cyclin A by PI3K/AKT pathway, ultimately promoting granulosa cell proliferation [186].

### FoxO signaling pathway

Forkhead box O (FoxO) is a family of transcription factors consisting of FoxO1, FoxO3, FoxO4 and FoxO6. OS can activate FoxO signaling pathway through phosphorylation, mono-ubiquitination and glycosylation [187]. Activated FoxO is transferred to the nucleus and regulates the expression of a series of downstream genes, further regulating apoptosis, autophagy and cell cycle arrest [188].

FoxO1 has been extensively studied in ovarian aging. It was confirmed that FoxO1 expression was most abundant in the granulosa cells of atretic follicles and was mainly localized in the nucleus [189]. Liu et al. found that 3-NP intraperitoneal injection significantly promoted FoxO1 transfer into the nucleus, upregulated the expression of PUMA, induced granulosa cell apoptosis and eventually led to follicular atresia [190]. In vitro experiments further showed that H_2_O_2_ activated the FoxO1 pathway and promoted the expression of downstream FasL, CASP3, and Bim, thus leading to apoptosis in the ovarian granulosa cells in a dose-dependent manner [191].

FoxO1 is also involved in the majority of the regulatory processes of FSH on granulosa cells [192]. FSH was previously shown to block post-translational modification of FoxO1 in granulosa cells and reduce FoxO1 expression, thus promoting cellular differentiation and follicle growth [189]. Furthermore, FSH inhibited the production of acetylated FoxO1 and its interaction with autophagy-related gene (Atg) protein through the PI3K/AKT pathway, thus reducing the level of cellular autophagy and ultimately inhibiting the death of ovarian granulosa cell [193].

FoxO3a is considered to be an ideal candidate gene for longevity and health [194]. The absence of FoxO3a led to the premature depletion of primordial follicles [195]. Cisplatin intervention significantly downregulated p-FoxO3a and antioxidant enzyme expression in mouse ovaries, impaired mitochondrial function, and ultimately accelerated apoptosis [196, 197]. A maternal high-fat diet could also reduce the number of primordial follicles in the ovaries of offspring via the FoxO3a pathway [198]. Conversely, oyster peptides upregulated the expression of FoxO3a and T-SOD, downregulated the expression of p53 and Bad, and attenuated D-galactose-induced premature ovarian decline [199].

### Klotho signaling pathway

Klotho is known to play an important role in the inhibition of aging. High levels of Klotho expression can prolong human lifespan, while low expression levels can lead to accelerated aging and increase the risk of multisystem diseases [200]. Klotho is mainly present intracellularly in transmembrane and secreted forms [201]. The transmembrane form of Klotho is a full-length transcript encoding 1014 amino acids. Once the short transmembrane structural domain is removed, this fragment can be released into the circulation in a secreted form [202].

Membrane Klotho is a co-receptor for endocrine fibroblast growth factors (FGF) and is involved in the activation of FGF receptors. Numerous studies have shown that the FGF-Klotho endocrine system plays a key role in the development of reproductive disorders by regulating the HPO axis [203]. Klotho-deficient mice exhibited dysfunction of the HPO axis as evidenced by a decrease in FSH and LH, follicular arrest in proestrus, gonadal atrophy and infertility [204].

Secreted forms of Klotho are more abundant than membrane Klotho. As an endocrine regulator, Klotho is thought to inhibit OS and modulate ion channel activity to exert positive anti-aging effects [205]. Studies have demonstrated that reduced Klotho expression activates the PI3K/AKT pathway, downregulates intracellular FoxO3a expression, disrupts oxidative homeostasis and inhibits autophagy, thus accelerating apoptosis [206].

## Additional antioxidant therapy that can improve ovarian aging

### Melatonin

Melatonin is an indoleamine secreted by the pineal gland located in the third ventricle. With lipid-soluble and water-soluble properties, melatonin can pass through cell membranes easily and is thus present in the blood and body fluids abundantly [207]. It has been found that melatonin is present in high concentrations in human follicular fluid [208]. Melatonin has a good antioxidant effect by neutralizing free radicals and increasing the activity of antioxidant enzymes such as SOD and GSH-Px [209]. As an important endogenous antioxidant, its antioxidant properties are superior to conventional antioxidants, such as vitamin C and E [210].

Melatonin is widely used in ART [211]. In patients undergoing IVF-ET, exogenous melatonin supplementation significantly reduced 8-OhdG and HEL concentrations in follicular fluid [212]. Additional melatonin also increased follicular growth rate, improved oocyte quality, increased fertilization rate and the number of quality embryos, ultimately increasing pregnancy rate [213]. Melatonin has also been shown to protect luteal granulosa cells from OS damage and increase progesterone secretion [214]. Moreover, melatonin was shown to reduce excessive Ca^2+^ levels in immature human oocytes during in vitro maturation (IVM) and improved the maintenance of mitochondrial membrane potential, thereby avoiding further ROS production [215].

Numerous experimental studies have demonstrated the important role of melatonin in improving oocyte quality and pregnancy outcomes. Melatonin significantly reduced the production of mitochondria ROS (mROS) in rat ovaries, increased telomere length, improved oocyte quality and ultimately increased the litter size [216]. Melatonin enhances the repair of DSBs via the non-homologous end joining (NHEJ) pathway to protect oocytes from the accumulation of DNA damage [89]. Melatonin can also upregulate antioxidant enzyme expression by inducing demethylation of the promoter regions of SOD, GSH-Px4 and CAT, thereby enhancing the antioxidant capacity of cumulus cells [217]. Melatonin activates ErbB1 and ErbB4 gene expression to promote embryo implantation and blastocyst growth, and further protects embryos from OS [218].

Melatonin has been shown to protect the ovaries from chemotherapy-induced damage. Barberino et al. performed melatonin pretreatment in mice prior to exposure to cisplatin. The results showed that melatonin reduced ROS levels in the ovary, increased mitochondrial activity, inhibited CASP3 expression and alleviated oocyte retraction and lysis [219]. Similarly, Jang et al. demonstrated that melatonin reduced cisplatin-induced excessive activation of the primordial follicles by modulating the PTEN/AKT/FOXO3a pathway [220].

### Vitamins

Vitamin C (VC), also known as ascorbic acid, is an excellent water-soluble natural antioxidant [221]. Extensive studies have demonstrated that VC can protect ovaries from harmful compounds such as NaF and As2O3 by upregulating the expression of SOD, CAT and GSH-Px and by reducing lipid peroxidation (LPO) [222]. Gai et al. found that VC also significantly ameliorated ambient aerosol fine particulate matter (PM2.5)-mediated ovarian damage, reduced the levels of oxidative products and inflammatory factors, decreased apoptosis and protected the mitochondrial ultrastructure [223]. In addition, VC could regulate cell proliferation and differentiation and steroid production, promote follicle growth and regulate endocrine secretion, and ultimately improve ovarian aging [224, 225].

Vitamin E (VE) is an important fat-soluble antioxidant [226]. A clinical study found that VE combined with selenium supplementation significantly improved AMH index, antral follicle count (AFC) and mean ovarian volume (MOV) in patients with occult POI [227]. Experimental studies have found that VE intervention can enhance the antioxidant capacity of ovarian tissue, regulate endocrine hormones, reverse follicular atresia, and restore the normal vascular distribution of ovarian tissue [228]. VE was also shown to be involved in the normal antioxidant function of GSH-Px1 as an essential cofactor [229]. In addition, VE can improve glucose uptake by follicular cells through the upregulation of glucose transporter-1 (GLUT-1) expression [230] and inhibit the abnormal proliferation of ovarian theca-interstitial cells [231], thereby protecting ovarian function.

### Stem cell therapy

Stem cells are a class of multipotential cells that can self-replicate and can differentiate into cells with multiple functions under certain conditions [232]. According to their differentiation potential, stem cells can be classified into totipotent stem cells, pluripotent stem cells and unipotent stem cells [233]. Due to excellent self-replication and multi-directional differentiation ability, stem cell therapy has attracted much attention in ovarian aging over recent years [234].

Mesenchymal stem cells (MSCs) are one of the most widely studied pluripotent stem cells. MSCs derived from different sources such as bone marrow, fat and amniotic fluid have been found to play a key role in restoring ovarian function and reproductive potential [235]. In the ovary, stem cells migrate mainly to the hilum and medulla after transplantation, with a few migrating to the cortex [236]. The migration of stem cells can exert effects on cell OS, proliferation and apoptosis mainly via paracrine action, thus repairing damaged ovaries [237]. Research has shown that human placental mesenchymal stem cells (hPMSCs) secreted a large amount of epidermal growth factor (EGF) which further activated the Nrf2/HO-1 signaling pathway to promote granulosa cell proliferation and oocyte maturation [238, 239]. Human umbilical cord mesenchymal stem cells (hUCMSCs) have been shown to reduce the autophagy level of theca interstitial cells (TICs) through the AMPK/mTOR signaling pathway, attenuate ovarian cell apoptosis, and ultimately improve ovarian function in POI rats [240]. It has been demonstrated that transplanted MSCs are able to restore ovarian function, including reducing OS damage, and participating in follicular maturation [241]. These findings provide new insights into our understanding of stem cell therapy and provide new avenues for developing more effective anti-aging treatments.

### Antioxidant monomers

Extensive research has demonstrated that a variety of monomers can protect ovarian function through antioxidant mechanisms. Tea polyphenols (TPS) is the general term for the polyphenols in tea leaves and it has been widely investigated for its important healthcare effects [242]. TPS has been shown to inhibit the surge of OS and relieved autophagic pressure in ovarian tissue caused by industrial plasticizers [243]. Grape seed proanthocyanidin extract (GSPE) is an excellent oxygen free radical scavenger [244]. GSPE exerts protective effects on both D-gal-induced ovarian hypofunction and natural ovarian aging. It can maintain the balance between cell proliferation and apoptosis, and reduce nuclear chromatin clumping in ovarian granulosa cells [245]. Resveratrol (Res) is a natural plant-derived phenol with excellent antioxidant activity [246]. Res has been found to attenuate the damage caused by cyclophosphamide on ovarian function, including reducing OS damage, inhibiting apoptosis and promoting ovarian stem cell repair [18]. In vitro studies have also revealed that Res can activate Nrf2 protein expression and reverse H_2_O_2_-induced OS damage in oogonial stem cells in a dose-dependent manner [18]. Curcumin is a diketone compound with excellent antioxidant and anti-inflammatory effects [247]. Curcumin intervention is shown to upregulate the expression of growth differentiation factor-9 (GDF-9) and bone morphogenetic protein 15 (BMP-15) and increase the number of follicles in mouse ovaries [248].

### Traditional Chinese medicine

Traditional Chinese Medicine (TCM) has a long history in the treatment of ovarian aging. Several TCM therapies have been demonstrated to modulate ovarian aging by alleviating OS damage. Kuntai capsule is a proprietary Chinese medicine that is widely used to treat menopause syndrome [249]. Research has shown that Kuntai capsule can upregulate SOD2, reduce ovarian apoptosis and follicular atresia, and increase AMH level in the ovaries of superovulated mice [250]. Bu Shen Huo Xue Tang (BSHXT) is a TCM formula that is clinically used in the treatment of POI. Research has shown that BSHXT can upregulate the expression of antioxidant enzymes SOD, HO-1 and NQO1 by activating the Nrf2/Keap1 signaling pathway and increase the levels of AMH and E_2_ in POI mice [251]. Acupuncture is an important external TCM treatment that has been proven to treat multiple age-related diseases [252]. Transcutaneous electrical acupoint stimulation (TEAS) is a combination of acupoint stimulation and electrical stimulation. TEAS intervention has been shown to upregulate the expression of antioxidant enzymes and proliferating cell nuclear antigen (PCNA), reduce apoptosis, and inhibit the loss of primordial follicles in ovarian senescent mice [253].

In conclusion, additional antioxidant therapy can improve the redox imbalance in the body, as summarized in Table 2. Some of the existing antioxidants, such as melatonin and vitamin E, have been used clinically and have proven their excellent antioxidant efficacy. However, it should be noted that most of the current antioxidant monomer studies have been applied only in animal models. Moreover, the efficacy of stem cell therapy in antioxidant therapy is also gaining attention. Therefore, these therapies should be validated in clinical settings in the future.

**Table 2:** Additional antioxidant therapy improving ovarian aging

Antioxidant Therapy	Therapeutic Effects Summary
Melatonin	• Increased fertilization rate, number of quality embryos and pregnancy rate • Acceleration of follicle growth, improvement of oocyte quality and increase of progesterone production • Improvement of primordial follicular hyperactivation • Neutralization of free radicals and increase of antioxidant enzymes activity • Enhancement of DSBs repair • Improvement of mitochondrial membrane potential • Increased telomere length • Inhibition of cell apoptosis
Vitamin C	• Reduced accumulation of OS damage products and inflammatory factors • Increase of antioxidant enzyme expression • Regulation of cell proliferation and differentiation • Promotion of steroid production
Vitamin E	• Assistance of GSH-Px1 to exert antioxidant function • Upregulation of GLUT-1 expression • Inhibition of abnormal proliferation of TICs
Stem cell therapy	• Secretion of large amounts of EGF • Promotion of granulosa cell proliferation and oocyte maturation • Reduced autophagy in TICs
Antioxidant Monomer	• Antioxidant and anti-inflammatory effects • Maintenance of homeostasis between cell proliferation and apoptosis • Reduced condensation of nucleus chromatin • Promotion of damaged ovarian stem cell repair
TCM related	• Reduction of cell apoptosis • Decreased follicular atresia • Upregulation of antioxidant enzyme expression • Inhibition of primordial follicles loss

*DSBs* DNA double-strand breaks, *OS* Oxidative stress, *GSH-Px* Glutathione peroxidase, *GLUT-1* glucose transporter-1, *EGF* Epidermal growth factor, *TICs* Theca interstitial cells, *TCM* Traditional Chinese Medicine

## Conclusion and future directions

The role and mechanisms of OS in ovarian aging have been extensively studied. Multiple factors, such as aging, smoking, and high sugar diets, can promote an OS state in the body; these factors could further accelerate ovarian aging via several key mechanisms, including apoptosis, increased inflammation and mitochondrial damage. The regulation of Nrf2, Sirt, MAPK, AKT and other OS signaling pathways play important roles in ovarian OS damage. Related antioxidants, such as melatonin and vitamin E, could improve OS status to restore ovarian function and therefore have potential clinical applications. There are still several aspects that need to be studied in the future: (1) multiple proteins and pathways are involved in the regulation of OS; the regulatory networks and key mechanisms of OS in ovarian aging still need to be studied in depth; (2) the majority of OS-related studies have focused on in vivo and in vitro experiments; further extension of these findings to clinical trials is needed to explore the safety and efficacy of antioxidant therapies; (3) antioxidant therapy has been initially proven to improve the quality of oocytes in vitro; further studies are now needed to determine whether it can improve pregnancy outcomes in patients undergoing ART.

## Data Availability

Not applicable.

## Abbreviations

4HNE: 4-Hydroxynonenal
AGEs: Advanced glycation end products
AKT: Protein kinase B
AMH: Anti-Müllerian hormone
ART: Assisted reproductive technology
Bax: Bcl-2-associated x
Bcl-2: B-cell lymphoma-2
CAT: Catalase
COH: Controlled ovarian hyperstimulation
DMF: Dimethyl fumarate
DSBs: DNA double-strand breaks
E_2_: Estradiol
ERK: Extracellular regulated protein kinases
FoxO: Forkhead box O
FSH: Follicle stimulating hormone
GSH: Glutathione
GSH-Px: Glutathione peroxidase
HO-1: Hemeoxy genase-1
HPA: Hypothalamic–pituitary–adrenal
HPO: Hypothalamic-pituitary-ovarian
IVF-ET: in vitro Fertilization-embryo transfer
JNK: C-Jun N-terminal kinase
LH: Luteinizing hormone
LPO: Lipid peroxidation
MAPK: Mitogen-activated protein kinase
MDA: Malondialdehyde
MSCs: Mesenchymal stem cells
mtDNA: Mitochondrial DNA
mTOR: Mammalian target of rapamycin
NF-κB: Nuclear factor-k-gene binding
NLRP3: Recombinant NLR family pyrin domain containing Protein 3
NQO1: NAD(P)H: nicotinamide adenine dinucleotide phosphate
Nrf2: Nuclear factor E2-related factor 2
OS: Oxidative stress
PI3K: Phosphatidylinositol 3-kinase
POI: Premature ovarian insufficiency
ROS: Reactive oxygen species
Sirt: Sirtuin
SOD: Superoxide dismutase
TCM: Traditional Chinese Medicine
